# Design Strategies for Large Current Density Hydrogen Evolution Reaction

**DOI:** 10.3389/fchem.2022.866415

**Published:** 2022-04-08

**Authors:** Lishang Zhang, Zhe Shi, Yanping Lin, Fali Chong, Yunhui Qi

**Affiliations:** ^1^ School of Physics and New Energy, Xuzhou University of Technology, Xuzhou, China; ^2^ School of Material and Chemical Engineering, Xuzhou University of Technology, Xuzhou, China

**Keywords:** electrochemical hydrogen evolution, electrochemical catalyst, hydrogen evolution reaction, intrinsic activity, architecture design

## Abstract

Hydrogen energy is considered one of the cleanest and most promising alternatives to fossil fuel because the only combustion product is water. The development of water splitting electrocatalysts with Earth abundance, cost-efficiency, and high performance for large current density industrial applications is vital for H_2_ production. However, most of the reported catalysts are usually tested within relatively small current densities (< 100 mA cm^−2^), which is far from satisfactory for industrial applications. In this minireview, we summarize the latest progress of effective non-noble electrocatalysts for large current density hydrogen evolution reaction (HER), whose performance is comparable to that of noble metal-based catalysts. Then the design strategy of intrinsic activities and architecture design are discussed, including self-supporting electrodes to avoid the detachment of active materials, the superaerophobicity and superhydrophilicity to release H_2_ bubble in time, and the mechanical properties to resist destructive stress. Finally, some views on the further development of high current density HER electrocatalysts are proposed, such as scale up of the synthesis process, *in situ* characterization to reveal the micro mechanism, and the implementation of catalysts into practical electrolyzers for the commercial application of as-developed catalysts. This review aimed to guide HER catalyst design and make large-scale hydrogen production one step further.

## Introduction

As the global fossil energy crisis and the greenhouse effect intensify, it is imperative to reduce the use of fossil fuels and explore alternative clean and sustainable energy sources (Lu et al., 2019; LunaDe et al., 2019; Wu et al., 2021). Hydrogen energy is considered one of the cleanest and most promising alternatives to fossil fuel because the only combustion product is water (Dunn, 2002). The blueprint of the hydrogen economy envisages that hydrogen is produced by water electrolysis through intermittent electric energy sources such as solar, wind, and tidal energy, which is then converted into usable electric energy in fuel cells or burned in engines (Chu and Majumdar, 2012; Jiao et al., 2021). However, so far, water catalysis develops slowly in the industry due to the expensive and unsatisfactory activity of noble metal catalysts (Wen and Guan, 2019). Although a range of non-noble metal electrocatalysts and catalyst design strategies have been developed, most have focused mainly on small current densities (<100 mA cm^−2^) which do not meet the requirements for commercialization (Jing et al., 2018; Wang et al., 2021a). For large-scale industrial hydrogen production, high current density (proton exchange membrane >1,000 mA cm^−2^, alkaline electrolytic cell >500 mA cm^−2^) and durability (>100 h) are crucial (Luo et al., 2019; Sen et al., 2020). Therefore, the development of robust hydrogen evolution catalysts with high current densities and durable catalytic time for industrial large-scale hydrogen production has greatly promoted the development of laboratories to commercial application.

Typically, hydrogen and oxygen are produced from the decomposition of water by two half-reactions, the cathodic hydrogen evolution reaction (HER) and the anodic oxygen evolution reaction (OER) (Tasneem and Abbasi, 2011; Zhu et al., 2017). Currently, platinum group metal-based catalysts show the best HER catalytic activity, but their rarity and expensiveness hinder their large-scale applications, resulting in hydrogen production only accounting for a small fraction (about 4%) of the total hydrogen production (Zou and Zhang, 2015). Therefore, it is highly desirable to develop HER catalysts based on non-noble metals and have an outstanding activity and durable long-term stability at large current densities (Christiane et al., 2018; Zhang et al., 2020a). Earth-abundant transition metal-based nanomaterials are considered promising electrocatalysts due to their low cost and high catalytic performance (Xie et al., 2021; Yu et al., 2021). In the past few years, a large number of promising catalysts have been explored, designed, and evaluated. However, many previous catalysts are still unsatisfactory in activity and stability, and require further research (Yang et al., 2021; Zhang et al., 2021). In addition, there are few reports on the efficient and stable operation of catalysts at industrial large current densities (Qian et al., 2021). Furthermore, some other obstacles, such as the bulk preparation strategies, the wreck and detach of active materials during the catalytic process, and the accumulation and the growth of bubbles on the catalyst surface, hinder the commercial application (Xu et al., 2021).

Here, we provide HER catalysts with an overview of exciting recent advances in efficient electrocatalysts with performance comparable to expensive noble metal-based catalysts. Then design strategies for the intrinsic activity and architecture design, including superaerophobicity, superhydrophilicity, and adaptability, are discussed. Finally, challenges and prospects for performance-oriented design rules that guide high-strength, durable HER electrocatalysts/electrodes at large current densities are presented.

## Design Strategies

**FIGURE 1:**
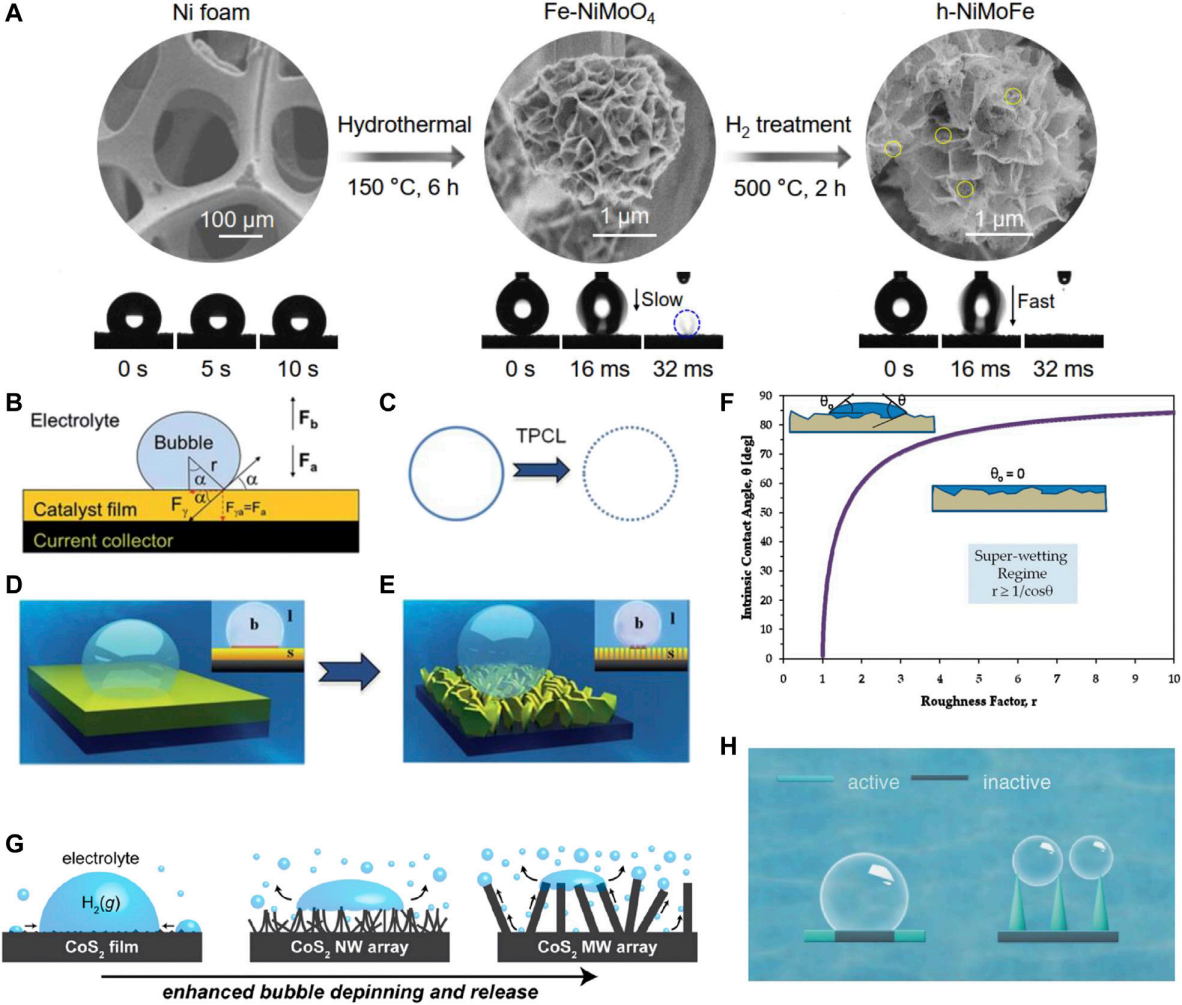
**(A)** Synthesis of the h-NiMoFe catalyst and wettability characterization on different samples, reproduced with permission from Luo et al. (2021). **(B)** Force analysis of a single bubble on the catalyst film; **(C)** triple-phase contact lines (TPCLs) on different electrode states: flat (left) and nanostructured (right) **(D,E)** schematic illustration of the adhesion behavior of bubbles on different electrode states: flat film (left) and nanostructured film (right), reproduced with permission from Lu et al. (2015). **(F)** Minimum values of the roughness coefficient necessary to facilitate complete diffusion of the liquid on the surface, reproduced with permission from Drelich and Chibowski (2010). **(G)** Schematic diagram of the evolutionary behavior of bubbles formed on CoS_2_ films with different surface structures, reproduced with permission from Faber et al. (2014). **(H)** Schematic illustration of bubble and catalysts contact, reproduced with permission from Xie et al. (2021).

So far, many high-performance water electrolysis catalysts have been developed, but most of them are operated at small current densities (<100 mA cm^−2^), which is far from the industrial requirement (Geng et al., 2021; Wang et al., 2021b). In addition, most of these catalysts are in the powder form, and the active center may detach from the electrode when expelling violent bubbles, requiring frequent replacement of the catalytic material in actual high current density industrial production (Zhang et al., 2018; Sun et al., 2020). Even if supporting materials are developed, few catalysts have been tested at high current densities. Therefore, the development of robust catalysts with high current density is particularly important from the perspective of economic benefits and applications. Luo et al. (2021) reported a hydroxide-mediated nickel-based catalyst for high-current density HER. The h-NiMoFe catalyst is loaded on a piece of Ni foam (NF) by a two-step method, as shown in Figure 1A, which delivers an impressively good performance that the current density is 1,000 mA cm^−2^ at a relatively low overpotential of 98 mV. According to their detailed microstructure characterization, the strong interactions between Ni and Mo/Fe could tailor the local electronic structure of Ni, and make hydroxide surface richer than other samples. As a result, even at high current densities, the h-NiMoFe catalyst could stabilize hydroxide on its surface. Impressively, the h-NiMoFe catalyst could be prepared on a meter scale, which has the prospect of industrial application. Zhang et al. (2020b) reported a fluorine-doped cobalt–iron phosphide supported on an iron foam (IF) catalyst. This F-Co_2_P/Fe_2_P/IF catalyst shows excellent HER activity that the overpotential is only 260.5, 292.2, and 304.4 mV at large current densities of 1,000, 2000, and 3,000 mA cm^−2^, respectively. Yu et al. reported a hierarchically structured 3D electrode fabricated by growing amorphous, mesoporous NiFe-LDH nanosheet network on a 3D MXene/NF frame (Yu et al., (2019a). This electrode was directly used as a binder-free catalyst which delivers a high current density of 500 mA cm^−2^at a low overpotential of 205 mV for hydrogen evolution.

During large-scale catalytic hydrogen evolution, massive hydrogen bubbles are rapidly formed at high current densities (Han et al., 2018). Bubbles accumulate on the contact surface of the catalyst and the electrolyte, which seriously hinders the mass transfer of the liquid, slows down the electron transfer, and reduces the exposed active sites number, resulting in decreased electrocatalytic activity and stability (Yu et al., 2019b). Thus, the challenge is separating the formed H_2_ bubbles to maintain the catalytic capacity of the electrodes in high current density industrial hydrogen production (Yang et al., 2019). It has been reported that “superaerophobic” surface structures can be assembled by forming array structures, which are essential for high-current HER since the superaerophobic surface could release forming bubbles in time (Lu et al., 2015). Since the accumulated bubbles on the surface could lead to the catalytic site blocking and the electrolyte diffusion suppression, the superhydrophilic electrodes are expected to promote the wettability between the catalyst and the aqueous electrolyte, and accelerate the separation of bubbles through superaerophobicity. For superaerophobicity, the three-phase interface of electrode–electrolyte–bubble is formed, as shown in Figure 1B (Lu et al., 2015). In large current density HER, the rapidly generated bubbles usually adhere to the electrode surface in large quantities and then cluster together to form a gas film, leading to the decreased active sites and hindering the diffusion from the electrolyte to the catalyst surface. It is indispensably needed to release the gaseous products from the electrode surface in time before bubble accumulation. Therefore, making the surface dislike the gaseous products beneficial for the bubble release, the catalyst surface engineering is thus of great prospective to solve this problem. Theoretically, the release diameter of the bubbles depends on the adhesion force of the catalyst film, and the adhesion force originates from the three-phase (solid, liquid, and gas) contact line (TPCL). As displayed in the left part of Figure 1C, the TPCL is a continuous circle when the electrode is flat. Constructing nanostructured electrodes provides a rougher surface, which significantly reduces the surface solid fraction and thus cuts the TPCL into discontinuous points (Figure 1C, right). Given that each point of the TPCL has the same adhesion force to bubbles of the same material, the broken TPCL shows a smaller cumulative adhesion force relative to the continuous TPCL. Therefore, the surface roughness is a key factor that influences the TPCL and adhesion force. Note that due to the interfacial energy balance, superhydrophilic surfaces are often superaerophobic; therefore, both design principles are often used simultaneously (Xu et al., 2021; GeorgeEaso et al., 2017). Superhydrophilic materials are textured and/or structured materials (rough and/or porous) with a surface roughness coefficient greater than 1, on which water (liquid) diffuses completely, as shown in Figure 1F, (Drelich and Chibowski, 2010). Roughness enhances the diffusion of the liquid and capillary forces control the wicking of the liquid into the textured material structure. Therefore, a common practical way to fabricate super wet surfaces is by manipulating the surface texture. For example, compared to nanosheets, vertically aligned nanoarrays (Figure 1D–Figure 1E (Lu et al., 2015)) can generate efficient gas escape, especially in high current densities; the three-phase (solid, liquid, and gas) contact line between the bubble and the electrode surface is in a discontinuous state, resulting in a particularly low contact area and low adhesion, similar to the microstructure of the bubble on the surface of a lotus leaf (Faber et al., 2014). For instance, Zhao and coworkers prepared a WS_2_ moiré superlattices electrocatalyst with both superhydrophilicity and superaerophobicity, which makes the big bubble split into small ones more naturally, maintains rapid and stable contact between the electrodes and electrolyte and deterring the formation of inactive sites (Figure 1H) (Xie et al., 2021). Wen and coworkers prepared a hierarchical amorphous CoMoSx electrocatalyst with both superhydrophilicity and superaerophobicity (Shan et al., 2020), which requires low overpotentials of 269 mV at 500 mA cm^−2^ for HER. The superhydrophilicity favors the entry of the electrolyte, and the superaerophobicity could facilitate the rapid departure of bubbles which accelerates mass transfer, especially at high current densities. Yin and coworkers obtained a nanovilli Ni_2_P electrode with superaerophobic and superhydropholic surfaces Yin et al. (2021). They found that these two characteristics can significantly facilitate mass and electron transfer, and the performance of the nanovilli Ni_2_P electrode is superior to that of the smooth Ni_2_P nanosheet array electrode, which is in accordance with the aforementioned theory (Figure 1F). Thus, the surface architecture design is an efficient way to not only enhance the superaerophobicity and superhydrophility, which are beneficial for the H_2_ release, but also increase the surface area which is conducive to the exposure of active sites and accelerate the mass transfer process of the electrolyte.

**FIGURE 2:**
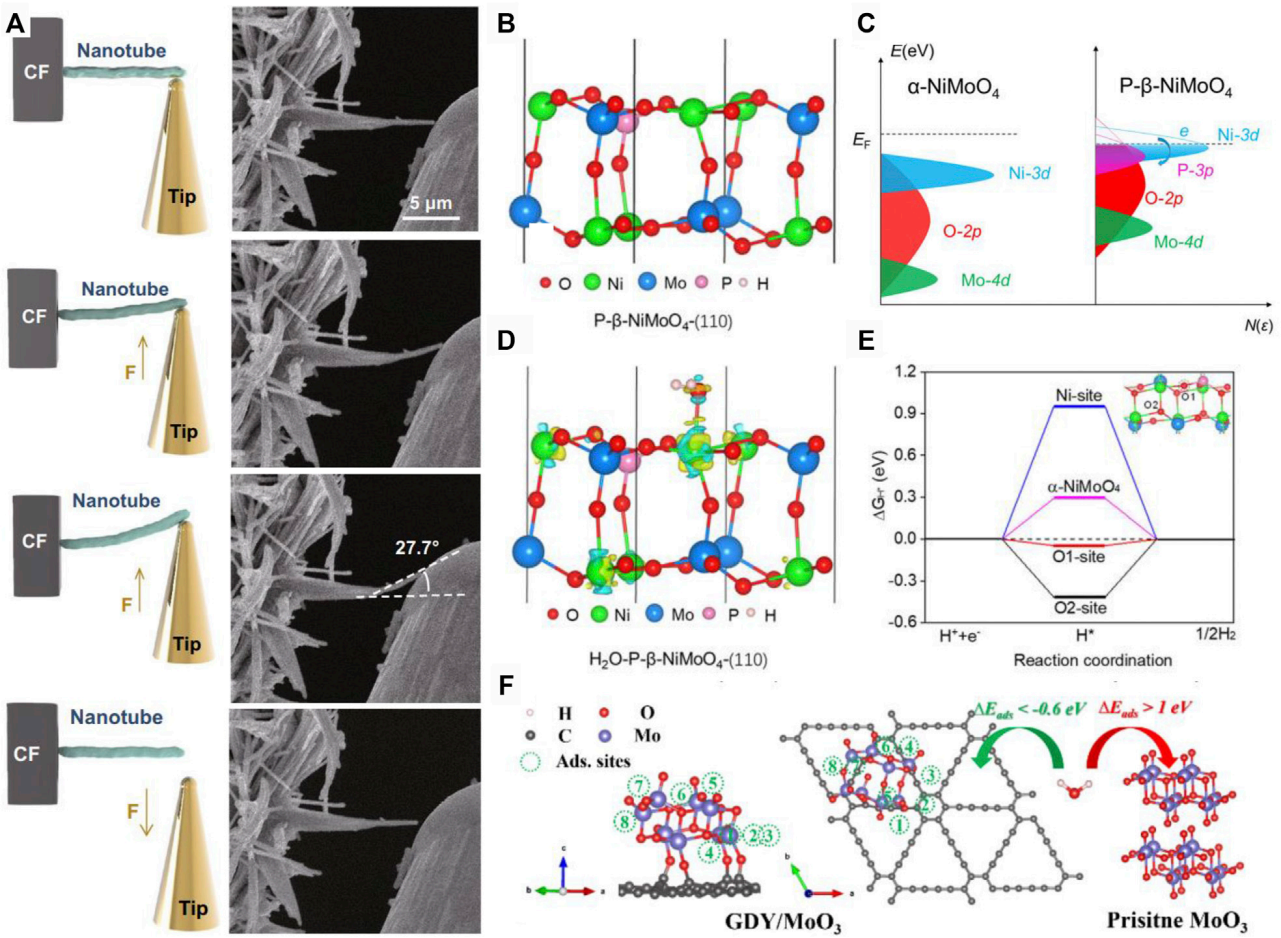
**(A)** *In situ* bending deformation and restoration measurement by SEM probe, reproduced with permission from Zhang et al. (2020c). **(B)** Optimized structure of P-β-NiMoO_4_-(110). **(C)** Active electric states in different phases of NiMoO_4_. **(D)** Charge density differences of H_2_O adsorbed on Ni sites in P-β-NiMoO_4_. **(E)** Hydrogen adsorption free energy (ΔGH*) in different exposed atoms in P-β-NiMoO_4_, reproduced with permission from Wang et al. (2021c). **(F)** H_2_O adsorption sites on GDY/MoO_3_ and H_2_O adsorption on pristine MoO_3_ and GDY/MoO_3_, reproduced with permission from Yao et al. (2021b).

In electrocatalytic processes, especially at high current densities, the tension and vibrational forces generated during bubble escape and collapse are widely regarded as important factors for poor stability (Zou et al., 2017). Therefore, high-current HER catalysts also require appropriate mechanical properties. From a machinery mechanics point of view, the gap-rich nanotubes interweave with stacked and interleaved nanosheets to form “springs” that can absorb vibrational wave energy, release rebound energy, and resist destructive stress from the surrounding environment (Bertolazzi et al., 2011; Liu et al., 2014). Zhang and coworkers reported a high-current HER catalyst 2D CoOOH sheet encapsulated Ni_2_P into tubular arrays Zhang et al. (2020c). They used *in situ* bending deformation and restoration measurement to detect the effect of mechanical toughness on the performance of high-current HER catalyst. The authors applied repeated bend-restoration tests to investigate the mechanical property. As shown in Figure 2A, a single nanotube of the Ni_2_P–CoOOH arrays was pushed by an SEM probe. As depicted, the maximum bending angle of a single nanorod is up to 27.7°. The nanotube can then restore to its original state, indicating its excellent mechanical stability. It was revealed that the high mechanical toughness of HER electrode material can buffer the electrolyte concentration polarization, accelerate hydrogen bubble rupture, and insure the long-term stability.

In a review article, it was noted that many of the reported catalysts improve their catalytic performance by increasing the mass loading or surface area of the catalyst and that the lack of an intrinsic catalytic activity center is a major barrier to the design and preparation of good catalysts (Jakob and Chorkendorff, 2019). High intrinsic activity is a prerequisite for high-current HER catalysts, which requires structural design at the atomic scale to tune the local electronic structure (Yu et al., 2021). To enhance the intrinsic activity, various strategies have been explored, such as phase engineering (Tran et al., 2016; Yao et al., 2021a), crystal facet engineering (Xi et al., 2021; Song et al., 2021), defect engineering (Ye et al., 2016; Yin et al., 2016), and polymetallic engineering (Li et al., 2021; Kumar et al., 2021). Wang and coworkers reported a strategy for achieving phosphate substitution and subsequent stabilization of the crystalline phase of metastable β-NiMoO_4_ (Figure 2B) Wang et al. (2021c). According to their study, compared to the α-NiMoO_4_ system, P-β-NiMoO_4_ contains optimized electronic states originating from Ni atoms near the Fermi level, which favors charge transfer from the active Ni to the surrounding atoms. In the β-NiMoO_4_ system, the promotion of the Ni-3d state after phosphate substitution favors the generation of the active electronic state. As a result, the adsorbed protons can readily accept electrons to produce hydrogen atoms, thus accelerating the whole HER process on P-β-NiMoO_4_. Phosphate substitution is proven to be imperative for stabilizing and activating β-NiMoO_4_, which can effectively generate the active electronic state and promote the intrinsic HER activity. In addition, simple perovskites have proven their HER ability during the past years with inferior activity to commercial Pt/C (Xu et al., 2016). Recently, Liu and coworkers used PrBa_0.94_Co_2_O_5+δ_ (PB_0.94_C) as a precursor for fabricating PB_0.94_C-based double/simple perovskite heterostructure (PB_0.94_C-DSPH) for HER Liu et al. (2021). Their research excludes the dominant effect of intrinsic activity for the simple perovskite phase on an outstanding performance of PB_0.94_C-DSPH. What is more, they prepared the catalyst by milling and calcination, which is promising for large-scale production. Another strategy is the discovery/design of new active sites with higher intrinsic activity (Nitish et al., 2022). Recently, Yao and coworkers reported an original 3D self-supporting graphdiyne/molybdenum oxide (GDY/MoO_3_) material. They introduced new intrinsic catalytic active sites (non-oxygen vacancy sites) by “sp C−O−Mo hybridization” on the interface (Figure 2F) Yao et al. (2021b). The GDY/MoO_3_ electrode displays excellent HER activity at high current densities, as the interfacial “sp C−O−Mo hybridization” facilitates electron transfer from GDY to MoO_3_, further leading to more efficient electron injection during HER (25-fold higher than MoO_3_) and decreasing the formation energy of oxygen vacancies.

## Conclusion and Outlook

The development of HER electrocatalysts with Earth abundance, cost-efficiency, and high performance for large current density industrial applications is of vital importance for H_2_ production. However, most of the reported catalysts focused mainly on small current densities (< 100 mA cm^−2^) which do not meet the requirements for commercialization. In this review, we recapitulated the exciting recent advances of effective electrocatalysts for HER whose performance is comparable to costly noble metal-based catalysts. Then design strategies with respect to the intrinsic activity and the architecture design are discussed. Although there are many large-current catalysts that have been developed, many challenges are still urgent to be overcome. 1) First, facile and scalable synthesis routes are urgently needed for the requirement of industry scale application of HER catalysts to be met (Zhang et al., 2020d; Qian et al., 2020). Among previously reported catalysts, NiMo-based electrodes have been demonstrated as the most active HER catalysts. As mentioned in this minireview, the h-NiMoFe catalyst shows excellent activity with a meter level synthesis, having the prospect of industrial application. The P-β-NiMoO_4_ shows superior performance than commercial Pt/C at large current densities as well. NiMo-based catalysts are often prepared by mild conditions, hydrothermal methods usually, making them promising for large-scale applications to meet the industrial dements. 2) Second, the phase characterization of catalysts is usually operated at their stable final states; *in situ* monitoring the phase information in the catalytic process is very important to reveal the micro mechanism of catalytic reaction (Jia et al., 2015). The mechanism of HER remains unclear and even controversial (Huang et al., 2021). In alkaline electrolytes, it is still under debate whether hydrogen binding energy acts as the only activity descriptor and whether other factors are the rate-determining steps (Zhang et al., 2019; Huang et al., 2021). Since the *ex situ* characterization could only detect the original state and final state after catalysis, the inside change cannot be observed. Therefore, the application of *in situ* and operational characterization under a real electrochemical process is very important to provide experimental evidence to determine key intermediates, and thus reveal the real phase-evolve process and the reaction mechanism. 3) Third, there is still a gap between academia and industry. In the actual efficiency first industrial testing process, test environment would be different: higher temperature, pressure, and electrolyte concentration. Therefore, the vital step is the implementation of catalysts into practical electrolyzers for the commercial application of as-developed catalysts. 4) Last, the stability is one of the most important parameters to evaluate the electrocatalysts for industrial applications. In acid electrolytes, non-noble metal-based catalysts usually show inferior stabilities since they are easily reacting with H^+^ in acid conditions. Therefore, no metal-based catalysts may become a promising candidate when applied at high current densities.
